# Refining the Nutrition Environment Measures Survey (NEMS) for Healthy Community Stores: Adaptations to Capture Alternative Food Retailers and Align with Dietary Guidelines

**DOI:** 10.3390/ijerph191912875

**Published:** 2022-10-08

**Authors:** Alex B. Hill, Ravneet Kaur, Samantha M. Sundermeir, Christina Kasprzak, Megan Winkler, Sara John, Rachael D. Dombrowski, Bree Bode, Joel Gittelsohn

**Affiliations:** 1 Detroit Food Map Initiative and Department of Urban Studies and Planning, College of Liberal Arts and Sciences, Wayne State University, Detroit, MI 48202, USA; 2 Division of Health Research and Evaluation, Department of Family and Community Medicine, College of Medicine, University of Illinois, Rockford, IL 61107, USA; 3 Department of International Health, Bloomberg School of Public Health, Johns Hopkins University, Baltimore, MD 21205, USA; 4 Department of Community Health and Health Behavior, University at Buffalo, Buffalo, NY 14260, USA; 5 Department of Behavioral, Social and Health Education Sciences, Rollins School of Public Health, Emory University, Atlanta, GA 30322, USA; 6 Center for Science in the Public Interest, Washington, DC 20005, USA; 7 Departments of Public Health and Kinesiology, College of Education, Health and Human Services, California State University San Marcos, San Marcos, CA 92096, USA; 8 Division of Kinesiology, Heath and Sport Studies, College of Education, Detroit, MI 48202, USA

**Keywords:** food access, healthy food retail, assessment, quantitative, index

## Abstract

Inadequate consumption of healthy food is an ongoing public health issue in the United States. Food availability measures of supply versus consumption of healthy foods are disconnected in many studies. There is a need for an objective assessment of the food environment in order to assess how the food supply aligns with the Healthy Eating Index (HEI). Data were collected as part of the Healthy Community Stores Case Study Project, including a refined Nutrition Environment Measures Survey for Healthy Community Stores (NEMS-HCS) and an updated Healthy Food Availability Index that aligns with the Healthy Eating Index (HFAHEI). This paper will focus on the NEMS-HCS development process, findings, and HFAHEI application. All food items were more likely to be found at grocery stores rather than corner stores. Food pricing was often above the Consumer Price Index averages for six food items. The NEMS-HCS assessment better aligned with the HEI because it included a wider variety of meats, frozen fruits and vegetables, and an increased selection of whole grains. HFAHEI scoring was inclusive of non-traditional and alternative community stores with a health focus, making it suitable for use at the local level, especially in neighborhoods where supermarkets and large chain stores are less common.

## 1. Introduction

Inadequate consumption of healthy food is an ongoing public health issue in the United States [1]. In 2015, few adults met the recommended fruit (12.0%) and vegetable (9.3%) intake [2]. Studies suggested that healthy food access is one of the predictors for increasing healthy food consumption [3,4,5] this two decades ago in their study where they found increased fruit and vegetable consumption by increasing the availability of it in their community food stores [5]. Different interventions [6,7] and national food and nutrition assistance programs such as the Supplemental Nutrition Assistance Program (SNAP) were implemented to address food security and improve food access for individuals and families with low income; however, the problem persists. This piqued interest among public health researchers of measuring the food environment, or the supply-side of food access, to examine the extent of the problem and impact of existing interventions and/or programs. Limited healthy food availability, in particular, can pose a challenge when trying to maintain a healthy diet that meets the Dietary Guidelines for Americans (DGAs) as measured by the Healthy Eating Index (HEI), specifically in low-income areas.

Previous studies have investigated the adequacy of the food supply at a national level [8,9] and found significant gaps between the optimal DGAs and the US food supply. In other words, there is disconnect with how the foods available within the food environment may or may not align with desired dietary guidance. For example, [9] found that the USDA’s tracking of 250 individual agricultural commodities analyzed against the DGAs revealed that the US food supply did not meet the demand for food categories like fruits, vegetables, and whole grains. The gaps noted included a lack of understanding of how supply levels across the food system translate to what is available to individuals and families at a local level, presenting an opportunity for local community store assessments. Local HEI scores are typically calculated from food frequency questionnaires or dietary recalls as a reflection of individual or population level dietary intake. However, there is opportunity to use retail food environment assessment tools, like an adapted Nutrition Environment Measures Survey for Stores (NEMS-S), to better characterize how the local food supply aligns with the HEI. 

Healthy food availability is typically measured in cities using retail food store counts by location where healthy foods are more likely to be stocked. However, these types of ecological measures only give a snapshot of the food environment and do not capture the dynamic nature of the local food supply [10]. Similarly, aligning food availability measures with supply and consumption of healthy foods is a critical disconnect in many food availability measures [8]. Lucan [10] provided clarity in showing that supermarkets carry a wide range of unhealthy products, yet are classified as “healthy.” The NEMS-S assessment tool has been the most commonly utilized as the basis for assessing the consumer food environment since it was developed [11,12]. The assessment tool captures multiple aspects of food availability related to healthy staple foods and beverages based on the most common food items purchased in the U.S., including availability, quality, and price. Pricing of healthy food is a similarly well-documented barrier to healthful eating [13,14,15,16].

Glanz et al. [12] found over 30 assessment tools that attempt to measure the retail food store environment. Notably, 4 of the 30 assessment tools were adaptations of the original NEMS-S. Since the time of Glanz et al.’s [12] review of assessment tools, the authors have identified four other new assessment tools including the Healthy Food Supply Score [17], SHELF Audit [18], AUDITNOVA [19], and a novel dietary assessment of online food retailers’ menus [20]. The NEMS-S is broadly based on the DGAs, but the food items assessed do not align comprehensively with the needs of an index like the HEI. The only other assessment tools that have attempted to closely align with dietary guidelines are the AUDITNOVA survey tool deployed in Brazil and the assessment of online food retailers in Ontario. Many of the tools identified by both Glanz et al. [12] and the authors adapted to accommodate small stores, healthy corner stores, or convenience stores, but in most instances, the assessment tool solution was to limit or reduce the food items and categories [17,21,22].

The NEMS-S was designed to be adapted and tailored for implementation in citywide assessments of a variety of food store types. Importantly, the categories included in this assessment tool can map onto HEI scoring categories, but require adjustment to map on directly. The NEMS-S sub-score for food availability, with added assessment categories, has been used as the primary quantitative data collection tool in the creation of a Healthy Food Accessibility Index (HFAI) in Baltimore [23]. The HFAI was first created in Baltimore based on NEMS-S assessments, but did not account for many of the amended food categories, such as frozen or canned fruits and vegetables or additional lean meats that align with the HEI score components and observed in various citywide NEMS-S projects [23,24,25,26,27]. Other limitations of the NEMS-S include a focus on brand name food items over lowest price food items, limited meat and seafood assessments, and less culturally inclusive grains like rice or tortillas. Expanded food categories are essential to better assess the true food availability at a neighborhood food store, but also to adequately assess linkages with food supply.

Given the need for an objective assessment of the food environment in order to assess how well the food supply aligns with the DGAs, the goals of this study were: (1) to create a version of the NEMS-S that aligns with the DGAs as measured by HEI to assess food access in community stores; (2) to develop a new scoring criteria for assessment and comparison of healthy community stores, and (3) to demonstrate the range of healthy food access (via an availability index and food pricing) in a sample of community food stores.

## 2. Materials and Methods

### 2.1. Overview

Data for the work presented in this paper were collected as part of the Healthy Community Stores Case Study Project. The project protocol with a detailed description of our methodology has been previously published [28]. In brief, a multiple case study approach was used to describe the experiences of seven healthy food stores in diverse urban settings. The inclusion criteria for store sites included: served a low-to-middle income urban region, had a clear mission to improve healthy food access, and demonstrated a capacity to carry out the research goals [28]. The choice of final stores was based on maximizing geographic and store type heterogeneity among a convenience sample. Case study sites were all non-chain, independently-owned community food stores located in: Baltimore, MD, USA; Boston, MA, USA; Buffalo, NY, USA; Detroit, MI, USA; Chicago, IL, USA; Minneapolis, MN, USA; and Washington, DC, USA. To maintain the anonymity of the participant store, we refer to each store by the city in which the store is located; however, this use should not be interpreted as a representation of all stores within the named city, but rather a presentation of information on the single store that data were collected at for this study.

### 2.2. Data Collection

A variety of data collection methods were used to develop a case report for each site. These included in-depth interviews with store management and staff, sales recall with store managers, document review (store reports, news articles, photos, and maps), and the NEMS for Healthy Community Stores (NEMS-HCS) assessment. In this paper, we focus on the NEMS-HCS development process and findings.

The NEMS-HCS assessment was built in Qualtrics and deployed at all seven participating sites. A training workshop was provided to all site leaders and other site team members during an online workshop on NEMS-S and food retail assessment implementation. The training was recorded to be delivered to additional research assistants and materials were made available in a shared folder hosted on the cloud. Assessments were checked for missing or unclear data points as they were entered in order to ensure quality of data collection. 

This study uses data collected at each site via the NEMS-HCS at three separate timepoints roughly one month apart. One site had two locations and only collected data at two timepoints. Data collection occurred in person using a tablet or smartphone to complete NEMS-HCS assessments. Store success and strategies for success emerging from in-depth interviews are captured in John et al. [29], also in this special issue. 

### 2.3. Development of the Food Environment Tool

A modified version of the NEMS-S was implemented in this study based on previous work in Detroit, Flint, and Chicago [24,26,27,30]. We call it the NEMS for healthy community stores (NEMS-HCS). The USDA’s HEI is the standard for measuring healthful eating by Americans while there is no standard measure for food availability. As a result, the NEMS-HCS was iterated to align with the HEI food categories. 

**Figure 1:**
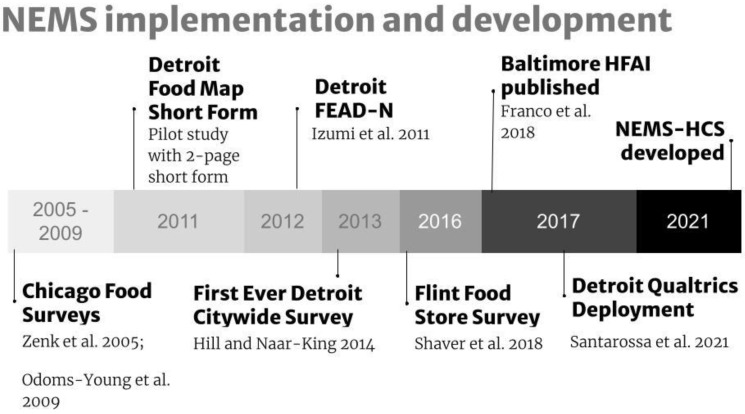
Timeline of food environment assessment instruments and their development. [4,23,24,26,27,30,31].

The NEMS-S tool has been used and adapted for multiple city surveys of food retail stores (See Figure 1). Early work in Detroit [26] developed a two-page short form based on the original NEMS-S eleven-page survey that was formatted for an outdated scanning machine for data entry and scoring. The frozen dinners category was dropped during this effort after a 2011 pilot project in Detroit found too much variability in frozen dinner offerings, resulting in 10 remaining food categories from the original NEMS-S. Pricing data collection remained unaltered from the original NEMS-S assessment with only related additions that mirrored existing price data collection criteria (e.g., chicken breast was added, and pricing was per pound, similar to other meat). During the same time period, food environment assessments were being tested in Chicago [24,31,32] and eventually, that knowledge came to Detroit in the form of the NEMS-based Food Environment Assessment for Diverse Neighborhoods (FEAD-N) survey tool [4]. The Detroit short form was deployed citywide in 2013 and again in 2015, before it was adapted for use in Flint after the water crisis that caused widespread lead poisoning, to include lead mitigating foods [27]. The NEMS-S was deployed with expanded fresh produce assessment in Baltimore where a Healthy Food Availability Index (HFAI) was first developed [23]. The NEMS-S tool was finally built within Qualtrics to reduce errors in data collection and aid quick analyses [30]. 

For this study, the 10 food categories used from the NEMS-HCS were matched to corresponding food item categories of the Healthy Eating Index (HEI) in order to assess opportunity or support of the DGAs (see additional information below). Categories included milk and dairy, fruits, vegetables, frozen fruits and vegetables, meat and protein, beverages, bread, and cereal (see Table 1). Availability and pricing data were collected for all categories. Quality was assessed for fresh fruits and vegetables. 

### 2.4. Availability Index Development

The Healthy Food Availability for Healthy Eating Index (HFAHEI) was developed as a part of this project in order to align the 13 HEI categories with 10 of the original 11 food categories assessed in the NEMS-S. HFAHEI aligns 10 food categories across the HEI and NEMS-S in order to get a snapshot of the local opportunity for healthy eating based on healthy food availability (see Table 1). This adapted food availability for healthy eating builds on previous work by [23] that utilized the NEMS-S availability sub-score and similar modified NEMS measures for healthy food availability by Campbell et al. [33] that focused on lowest price rather than brand name items. Scoring was informed by past work [27] to include more food items, such as yogurt and cheese in the dairy category that assist in mitigating lead absorption in the body. Shaver et al. [27] also informed the inclusion and scoring criteria for frozen fruits and vegetables based on Cavanaugh et al. [25] and the NEMS-S-Revised by the Rudd Center [34] NEMS for Corner Stores (NEMS-CS), as well as expanded protein food items. The application of scoring was based on the original criteria developed by Glanz et al. [11] expanded fruit and vegetable criteria from Franco et al. [23] frozen fruits and vegetables from Cavanaugh et al. [25] and the additional dairy, protein, and beans items adapted by Shaver et al. [27]. 

### 2.5. Food Pricing

Pricing data were also collected as part of the NEMS-HCS from all the participating food retailer locations and were analyzed against the Consumer Price Index (CPI) for selected items in order to compare how case study sites followed national trends in affordable food pricing. Food items from the CPI were limited to healthy foods and food items that were assessed as part of the NEMS-HCS. Five food categories from the NEMS-S matched six available food items in the CPI for comparison. The food items for comparison included: low-fat milk, apples, bananas, tomatoes, chicken breast, and wheat bread. As the NEMS-HCS was predominantly implemented over the course of three months (May–July) in 2021, the case study sites’ food prices were averaged across those months. Similarly, a three-month average of the CPI was pulled for comparison, matching the timeframe of the data collection.

### 2.6. Analysis

#### 2.6.1. Availability Index

Average HFAHEI scores across multiple observations from each site were created and compared across the study sites based on financial model (nonprofit, including co-op, or for-profit), store type (grocery or corner), and square footage. The HFAHEI was also compared to the standard HFAI based on the NEMS-S availability sub-score. 

#### 2.6.2. Food Pricing

Average food prices for each site were created and then compared to a three-month price average of the CPI products which matched the timeframe of the NEMS-HCS data collection. We also calculated values for the NEMS-S food price sub-score for each site. The original NEMS-S price sub-score is meant to compare pricing of healthful foods versus less healthful foods, such as wheat bread compared to white bread or lean meat compared to ground chuck beef.

## 3. Results

Data were collected from a total of 7 food stores serving neighborhoods who were largely low-income, African American, Indigenous and/or people of color [28]. The store sizes ranged from 900 sq ft to 65,000 sq ft with three stores categorized as ‘Grocery stores’, two as ‘Corner stores’, and two as ‘Supermarket/Market’ (treated as ‘Grocery’ for this paper). Further information about store characteristics and type of business model has been published [28]. Below, we present our results by describing the availability index and price comparison of available food across sites, store types, and square footage. 

### 3.1. Availability

The majority of food products assessed in the NEMS-HCS were available at 5/7 case study stores, as five stores demonstrated a score of 19 or higher on the HFAHEI. All food items were more likely to be found at grocery stores rather than corner stores. Most notably, meat, seafood, milk, and dairy availability were lacking at corner stores. Grocery stores similarly had a greater variety of fruits and vegetables available compared to corner stores. However, small format grocery stores stood up surprisingly well alongside large full-line grocery stores (See Table 2). Notably, the Washington, DC store, with a smaller square footage than the two corner stores in this study, scored just a single point lower than the full-line grocery store ten times its size in Boston, MA (HFAHEI 20.3 vs. 19.3 respectively). Many of the large point differences seen between the HFAI and HFAHEI were related to the inclusion of a wider range of meat and protein alternatives included in the HFAHEI. 

The specific availability of fruits and vegetables are shown in Table 3 along with the scoring by site. The two top scoring stores earned full points for fruit and vegetable availability and were grocery store types, while the two lowest scoring stores had limited availability of fruits and vegetables, although notably less availability for fruits, and were corner stores. 

### 3.2. Index Comparisons

**Figure 2:**
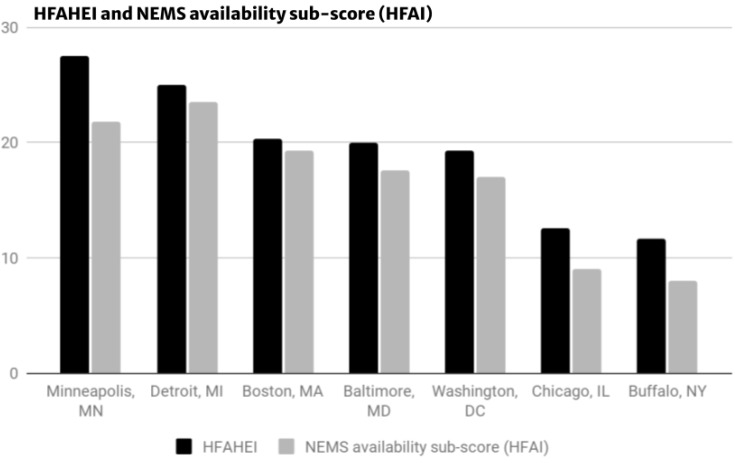
HFAHEI compared to HFAI scoring.

Figure 2 presents a comparison of the HFAHEI scoring and the NEMS pricing sub-score. The inclusion of additional food items in the HFAHEI most benefitted the study site in Minneapolis, MN, USA with a 5.7-point increase. The increased inclusion of healthful food items also benefitted corner stores which saw similar large point increases with a 3.6-point increase in Chicago, IL, USA and Buffalo, NY, USA. Traditional full-line grocery stores did not see major differences in point scoring between the HFAHEI and HFAI. 

The HFAHEI scores varied across financial model, store type, and square footage (See Figure 2) with store type between grocery versus corner stores demonstrating the greatest variance (an 8.7-point difference). 

**Figure 3:**
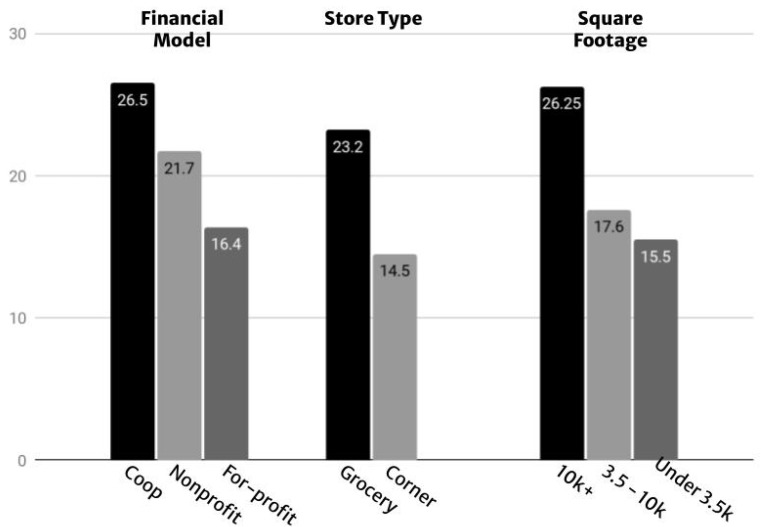
HFAHEI point scoring breakdowns by financial model, store type, and square footage.

Store square footage had a similar gap with an 8.65-point difference between stores over 10,000 square feet and stores between 3500 to 10,000 square feet (See Figure 3). Store financial models generated an interesting result with nonprofit stores scoring better collectively than for-profit stores. 

### 3.3. Price Comparison

For the six food items compared across the CPI and data collected at the study sites, many were priced higher than the average CPI price (Table 4). Notably, meat (chicken breast) was typically cheaper among grocery sites, except the non-profit, cooperative in Minneapolis, MN, USA, over corner store sites in this study. 

Almost all of the stores’ average market basket was above the CPI average, which corresponded with their NEMS-S price sub-score. Only the Detroit, MI store location scored positively for price comparison with the NEMS-S scoring, yet even then, the price score was very low. The NEMS-S sub-score could range from −6 to 16 points, and all but one store had a negative score, indicating pricing for healthful food was not ideal. 

## 4. Discussion

In this study, we explored and compared healthy food availability and prices across a diverse set of stores including for-profit, non-profit, and co-op. We found that grocery stores are more likely to stock more healthy food items than corner stores, specifically, meat, seafood, milk, and dairy products. Corner stores also had a lower availability of fruits and vegetables, including frozen fruits and vegetables, than full-line grocery stores. These findings are consistent with the previous studies, demonstrating corner stores as a potential target for intervention to improve the local/neighborhood food environment [11,25,35].

However, one corner store, i.e., Washington, DC, USA, scored higher for availability of food items irrespective of its much smaller store size. This highlights the importance of non-traditional business models, as the corner store at the Washington, DC, USA site was a social enterprise where community residents participated in the store decision-making [36]. Broadly, the grocery versus corner store results match what has been found by Cavanaugh et al. [25] in the Philadelphia Healthy Corner Store Initiative where meat and low-fat milk were often unavailable.

This is the first study, to our knowledge, to adapt a store food environment assessment tool to align with the HEI. Numerous studies, nationally and internationally, have adapted NEMS [12,37,38,39]. Yet, the underlying reason was to create a version of the tool that is more applicable to the context of the local U.S. urban populations. However, Borges et al., [19] developed Consumer Food Environment Healthiness Score (CFEHS) by adapting existing measurement tool named AUDITNOVA, following Dietary Guidelines for the Brazilian Population. The emergence of multiple new assessment tools since 2017 that are focused on small stores, corner stores, and/or alternative food retailers demonstrates the need for an assessment tool. Glanz et al. [12] found that the NEMS-CS had only been utilized in two studies and the authors identified an additional adapted version [22].The NEMS-S has served as an assessment tool baseline and therefore, makes the most sense to be a tool adapted to accommodate the DGAs and healthy community stores.

The creation of the HFAHEI improves assessment and comparison of non-traditional store types like cooperatives and corner stores by considering a wider range of more healthful food items. The inclusiveness of the HFAHEI for all types of community stores working to improve healthy food access in cities highlights the benefit of applying the NEMS-HCS. This novel adaptation filled the gap in assessment of how the local retail food environment aligns with the HEI. HFAHEI improves upon the original HFAI scoring by including more relevant food items to the HEI. The gap in assessing healthy food eating versus healthy supply is one that can now be surveyed using the NEMS-HCS to understand the opportunities in a low-income city for residents to access food that supports a healthy diet. 

This study found the adapted NEMS-HCS assessment better aligned with the HEI because it included a wider variety of meats, frozen fruits and vegetables, and an increased selection of whole grains. Further, the subsequent expanded HFAHEI scoring was more inclusive of non-traditional and alternative community stores with a health focus, making it suitable for use at the local level, especially in neighborhoods where supermarkets and large box stores are less common, and alternative stores such as those included in this large case study are more common [28]. The NEMS-HCS was able to build from previous work [24,26,27,30,34] to streamline food categories and food items that best represented a healthy community store. The HFAHEI scoring criteria similarly aggregated the best steps from previous work in order to emphasize healthfulness in assigning points and then compare stores for their healthfulness related to the HEI. 

The HFAHEI scoring difference between non-profit and for-profit financial (and ownership) models presents a relatively new area of research into alternative and non-traditional community food retail models. A minimum HFAHEI score of at least 20 points was found to be needed for a healthy community store. The HFAHEI scoring reiterated the importance of full-line grocery stores in supporting neighborhood health in terms of availability, but the price difference remained wide. Pricing data were collected during the COVID-19 pandemic and may be skewed as a result. The non-profit stores scored higher overall and this is likely due to more of the non-profit stores falling into the grocery store type, which tend to have a wider selection of healthy food options. The cooperative financial model in Minneapolis, MN was an outlier in that the store’s financial structure and values, such as sustainably grown and sourced products, dictated purchasing decisions that were nearly always priced higher than the CPI average [29]. However, this store also provided a needs-based program where low-income customers received a 10% discount at the checkout. Even smaller format stores with a health-focused message scored very high in availability, but also with higher-than-average pricing, which may be related to store–vendor relationships and a given store’s ability (or inability) to leverage lower prices through a network of multiple vendors [29]. 

The NEMS-HCS and its associated HFAHEI scoring could be used as a tool for communities to evaluate and improve food access in their neighborhoods. Community partnerships, such as those with academic institutions, can use this tool to assess local food environments and identify gaps in healthy food availability. The data collected could be used to present to policy makers to build a case for local policy change such as healthy checkouts and staple food ordinances, as it aligns with pre-established criteria—the HEI. This tool can also be used by retailers for goal setting related to stocking of healthy options and nutrition-focused provisioning guidelines. 

The USDA, in a 2022 request for comment on “small, mid-sized, and otherwise independent (SME)” processors (USDA 2022), highlights the recent consolidations in food retail with the potential to create unfair and anticompetitive practices throughout the food supply chain. This project has demonstrated the necessity and opportunity to provide healthy foods by small and mid-sized independent retailers [40]. The HFAHEI could be deployed to understand availability by competition in the market, ensuring the inclusion of small and mid-sized community stores. Assessing stores at the local level with the NEMS-HCS could similarly help in identifying gaps in stocking or food categories with commonly high prices as a method to target improvement. 

Both the NEMS-HCS and HFAHEI were applied in 7 store sites within cities, but have the potential to be tested citywide and across different types of food stores and geographies. The number of food store alternatives offers the opportunity to use the NEMS-HCS in many new ways. An important comparison will also be applying both tools across urban, suburban, and rural areas where healthy community stores exist, but are difficult to compare. 

### Limitations

The sample of stores in this case study was small and limited; however, it represents a useful spectrum of store types. While there was only a single store surveyed per city, the seven stores represent examples of similarly situated low-income neighborhoods across the country. 

There is opportunity to further expand the NEMS-HCS to include additional food items that fill out the HEI assessment categories. Notably, “seafood and plant protein” and “greens and beans” categories are under-represented. 

Further work is needed to improve the HFAHEI for CPI price comparison. The CPI had limited data availability making affordability of food items difficult to assess across 7 cities. An improved source of food pricing information may need to be identified for future studies. Analysis may be more easily completed for single city or single geographic regions as the NEMS-HCS prioritizes a range of specific healthful food items rather than a broad range of food items that may skew an assessment negatively for community stores with limited suppliers. 

The refined NEMS-HCS would also benefit from a larger testing and a validation study as this was an exploratory study.

## 5. Conclusions

The sustained and regular use of the NEMS-S assessment tool permits researchers to understand the food environment, health impacts, as well as make comparisons across locations and store type. The further adaptation of the NEMS-S to the NEMS-HCS in this study and the associated updated HFAHEI scoring to align with HEI criteria enhances the usefulness of the tool. The study results showed that full-line grocery stores scored higher overall and thus, remain critical in providing healthy food. However, community stores may benefit from non-traditional financial models, such as non-profits or co-ops, which scored the highest for both availability and affordability (John et al.). This adaptation of the NEMS-S could be used at the local level to identify gaps in the food supply and inform policy to support smaller food retailers with alternative business models in order to better align the food environment with the HEI. 

## Figures and Tables

**Table 1:** NEMS categories and food items aligned with Healthy Eating Index categories with point values and point scoring sources.

NEMS Food Categories and Food Items	HEI-2015 Food Category	Point Value	Point Scoring Source
(1) Milk and Dairy		___/6	
YES Low-fat/skim	Dairy	1	NEMS-S
Proportion ≥ 33%	Dairy	1	et al.
Proportion ≥ 50%	Dairy	2	NEMS-S
YES Lowfat cheese	Dairy	1	Shaver et al.
YES Lowfat yogurt	Dairy	1	Shaver et al.
(2) Fruit (fresh)		___/4	
0 varieties	Whole Fruits	0	NEMS-S
<5 varieties	Whole Fruits	1	NEMS-S
5–10 varieties	Whole Fruits	2	NEMS-S
10+ varieties	Whole Fruits	4	Franco et al.
(3) Vegetables (fresh)		___/4	
0 varieties	Whole Vegetables	0	NEMS-S
<5 varieties	Whole Vegetables	1	NEMS-S
5–9 varieties	Whole Vegetables	2	NEMS-S
10+ varieties	Whole Vegetables	4	Franco et al.
(4) Frozen Fruits and Vegetables		___/4	
0 varieties	Total Fruits	0	NEMS-CS
<5 varieties	Total Fruits	1	NEMS-CS
5–9 varieties	Total Fruits	2	NEMS-CS
10+ varieties	Total Fruits	4	NEMS-CS
(4) Meat and Protein		___/6	
YES lean ground meat	Total Protein Foods	1	NEMS-S
YES boneless, skinless breast	Total Protein Foods	1	Franco et al.
≥2 lean varieties	Total Protein Foods	1	Franco et al.
YES white fish	Seafood and Plant Proteins	1	Shaver et al.
≥2 varieties of non-meat proteins (canned/dried beans, eggs)	Greens and Beans	2	Shaver et al.
(8) Beverages		___/1	
YES 100% juice	Total Fruits	1	Shaver et al.
(9) Bread		___/4	
YES 100% whole wheat bread	Whole Grains	2	NEMS-S
>2 varieties wheat bread	Whole Grains	2	Franco et al.
(11) Cereal		___/2	
YES healthier cereal	Refined Grains	1	NEMS-S
≥2 low sugar varieties	Added Sugar	1	Franco et al.
TOTAL HFAHEI Score		30	

**Table 2:** HFAHEI (0–30) difference comparison with HFAI (−6–25) by store type, square footage, and financial model.

HFAHEI	NEMS Availability Sub-Score (HFAI)	Point Difference	City **	Type	Square Footage	Financial *
27.5	21.8	5.7	Minneapolis, MN, USA	Grocery	20,000	Nonprofit, Cooperative
25	23.5	1.5	Detroit, MI, USA	Grocery	65,000	For-profit
20.3	19.3	1.0	Boston, MA, USA	Grocery	3850	Nonprofit
20	17.6	2.4	Baltimore, MD, USA	Grocery	7000	Nonprofit
19.3	17	2.4	Washington, DC, USA	Corner	900	Nonprofit
12.6	9	3.6	Chicago, IL, USA	Corner	3500	For-profit
11.6	8	3.6	Buffalo, NY, USA	Corner	1400	For-profit

* Adapted from Gittelsohn et al. 2022 ** City names represent individual store locations of the 7 participating retailers.

**Table 3:** Fruit and vegetable availability by location with HFAHEI scores.

HFAHEI	Fruit ___/4	Vegetable ___/4	Frozen Fruit Vegetable ___/4	City *	Type	Square Footage
27.5	4	4	4	Minneapolis, MN, USA	Grocery	20,000
25	4	4	4	Detroit, MI, USA	Grocery	65,000
20.3	4	4	2	Boston, MA, USA	Grocery	3850
20	4	2	3	Baltimore, MD, USA	Grocery	7000
19.3	4	4	4	Washington, DC, USA	Corner	900
12.6	1	3	0	Chicago, IL, USA	Corner	3500
11.6	1	3	2	Buffalo, NY, USA	Corner	1400

* City names represent individual store locations of the 7 participating retailers.

**Table 4:** Consumer price index averages compared to individual store location price averages for select food items.

	CPI (May–July 2021)	Minneapolis, MN, USA **	Detroit, MI, USA	Boston, MA, USA	Washington, DC, USA	Baltimore, MA, USA	Chicago, IL, USA	Buffalo, NY, USA
**Type**		Non-profit, cooperative, Grocery	For-profit, Grocery	Nonprofit, Grocery	For-profit, Corner	Nonprofit, Grocery	For-profit, Corner	For-profit, Corner
**Milk and Dairy**								
Low-fat Milk (Gallon)	3.14	**+0.85**	**+0.15**	**+0.55**	---	−1.35	---	---
**Fruits**								
Apples	1.33	**+0.83**	**+0.16**	−0.04	**+0.26**	**+0.16**	**+1.17**	**+1.34**
Bananas	0.60	**+0.68**	**+0.09**	−0.21	**+0.39**	−0.14	**+0.40**	**+0.45**
**Vegetables**								
Tomatoes	1.82	**+1.89**	**+0.17**	−0.37	**+0.17**	**+1.03**	**+1.17**	−0.05
**Meat and Protein**								
Chicken breast	3.41	**+4.58**	−0.47	−0.67	**+0.76**	−0.92	**+1.58**	---
**Grains**								
Wheat bread	2.09	**+2.80**	−0.09	**+0.40**	**+1.90**	**+1.90**	−0.20	**+1.40**
**TOTAL**	12.39	**+11.63**	−0.01	−0.34	**+0.34**	−0.32	**+0.98**	−3.41
**Percent above average prices**	---	100%	66%	50%	---	33%	---	---
**NEMS Price Sub-score** **(−6–16)**	---	−1.00	0.66	−0.66	−2.00	−1.66	−1.00	−1.66

**Bolded** prices are above the CPI average ** City names represent individual store locations of the 7 participating retailers.

## Data Availability

The data presented in this study are available on request from the corresponding author. The data are not publicly available due to confidentiality considerations.
